# Financial Outcomes of Managed Entry Agreements for Pharmaceuticals in Italy

**DOI:** 10.1001/jamahealthforum.2023.4611

**Published:** 2023-12-28

**Authors:** Francesco Trotta, Maria Alessandra Guerrizio, Aurora Di Filippo, Agnese Cangini

**Affiliations:** 1 Agenzia Italiana del Farmaco, Rome, Italy; 2 Università Cattolica del Sacro Cuore di Roma, Rome, Italy

## Abstract

**Importance:**

Most countries in the Organisation for Economic Co-operation and Development apply managed entry agreements (MEAs), reimbursement arrangements between manufacturers and payers, to pharmaceuticals. Few data exist regarding their ability to lower expenditures.

**Objective:**

To analyze the financial outcomes of MEAs for pharmaceuticals from 2019 to 2021 in Italy.

**Design, Setting, and Participants:**

In this observational study of MEAs and pharmaceutical spending in Italy, medications that were monitored through individually collected data and generated paybacks from manufacturers during the 2019 to 2021 study period were included in the analysis. Payback data were collected through pharmaceutical spending monitoring activities conducted by the Agenzia Italiana del Farmaco (Italian Medicines Agency). Expenditure data were collected through the Italian Drug Traceability System. Products were categorized by type of MEA: financial-based, outcome-based, or mixed.

**Main Outcomes and Measures:**

The main outcome was median payback as a proportion of expenditure by category of MEA. Results were also provided by subtype: cost sharing or capping models for financial-based MEAs and risk-sharing or payment-by-result models for outcome-based MEAs. Mixed MEAs were considered when medications had multiple indications with different MEA types.

**Results:**

A total of 73 medications with MEAs generated a payback by manufacturers during the study period. Six were either not reimbursable or delivered within the Italian National Health Service, and 5 had incomplete data. Of the 62 medications analyzed, 24 (38.7%) had financial-based MEAs, 30 (48.4%) had outcome-based MEAs, and 8 (12.9%) had mixed MEAs. A total payback amount of €327.5 million was calculated during the 3 years, corresponding to 0.9% of the €41.1 billion of total expenditures for medications purchased by public health facilities in Italy. Financial-based MEAs returned the highest payback revenues, €158.1 million; the outcome-based MEAs and mixed MEAs generated smaller paybacks of €74.5 million and €94.9 million, respectively. Overall, the median proportion of payback to expenditure on the medications analyzed was 3.8%. For mixed MEAs, the payback-to-expenditure proportion was 6.7%; for outcome-based MEAs, 3.3%; and for financial-based MEAs, 3.7%.

**Conclusions and Relevance:**

This observational study found limited evidence that MEAs lower pharmaceutical expenditures. Determining criteria for prioritizing MEA use, identifying potential design changes, and improving implementation may be needed in the future.

## Key Points

**Question:**

Are managed entry agreements (MEAs) important for the sustainability of pharmaceutical spending, and what are the financial outcomes of MEAs?

**Findings:**

In this observational study of the financial outcomes of medicines with MEAs from 2019 to 2021 in Italy, the median proportion of payback to expenditure was 3.8%.

**Meaning:**

These findings suggest that MEAs have limited importance for managing pharmaceutical expenditures; thus, prioritizing MEA use, identifying potential design changes, and improving implementation are valuable considerations.

## Teaser

This observational study analyzes whether managed entry agreements lower pharmaceutical expenditures using individually collected data from 2019 to 2021 in Italy.

## Introduction

The introduction of novel and often expensive therapies poses important challenges for payers, especially when limited evidence of the added therapeutic value exists at the time of pricing and reimbursement decisions. A majority of countries in the Organisation for Economic Co-operation and Development (OECD) now apply managed entry agreements (MEAs).^1^ These agreements aim to guarantee both access to novel, potentially innovative high-cost medications and sustainability of the pharmaceutical expenditure for payers, while managing uncertainty around their clinical effectiveness, cost-effectiveness, and budgetary effect.^2^

An MEA is defined as an arrangement between a manufacturer and payer that allows access to a health technology provided specified conditions are met.^3^ There are 2 main types of MEAs: financial-based schemes and health outcome–based schemes.^4,5^ The former, which is the most frequently applied in Europe, considers only financial aspects of a medicine, regardless of the potential effect on health outcomes, and may take the form of price-volume agreements with or without ceiling discounts or dose/utilization capping. In the case of price-volume agreements without a ceiling, the price of a medicine is associated with the defined volume sold, and the price declines when volume increases. According to price-volume agreements with ceiling, a maximum volume/budget that may be sold is established, and if the sales volume or budget is exceeded, the pharmaceutical manufacturer usually reduces the price of the medicine (ie, discount) or pays back the amount of sales exceeding the agreed levels (ie, rebate). Health outcome–based MEAs generally take either of these 2 forms: (1) coverage with evidence development, in which reimbursement is contingent on the initiation of postlaunch evidence generation, and (2) performance-linked schemes, in which reimbursement of covered products is associated with measures of clinical outcomes in practice settings.^4,6,7,8,9^ Performance-linked reimbursement schemes aim to enhance the cost-effectiveness of a new technology in clinical practice and may have a role in the financial sustainability of pharmaceutical spending.^5^

Italy is one of the first countries in Europe to have adopted MEAs; the Agenzia Italiana del Farmaco (AIFA/Italian Medicines Agency) agreed on its first contract in July 2006.^10^ Agreements negotiated by AIFA and manufacturers are monitored either through data collection by setting up individual registries or routine sales data collection. Monitoring registries in Italy, set up before the establishment of AIFA, were conceived to promote appropriate use of new medications and evolved both in terms of scope and technical features. They were initially established to confirm the risk-benefit profile of medications used in clinical practice (eg, cholinesterase inhibitors to treat Alzheimer disease through the Cronos project).^11^ Throughout several years, the scope of registries has expanded to include data collection on patient characteristics (eg, prognostic factors for treatment response) and long-term data on efficacy and safety as a condition for full access to pharmaceuticals reimbursed by the Italian National Health Service (INHS) (eg, the PSOCARE project).^12^ More recently, registries have been used to manage novel oncologic medications by associating access to novel therapies with economic sustainability (ie, payment-by-result model).^13,14^

Although MEAs have been used increasingly in the last decade in several jurisdictions, the body of current evidence on financial outcomes of MEAs is sparse.^15^ Published studies^16,17,18,19^ provided only aggregated data on revenues paid by manufacturers in Italy in a single year; however, these studies provided no economic details by MEA type or information on expenditure for medications under MEAs. Another study estimated savings generated by the application of an MEA related to only 1 medicine, which was signed by the Catalan Health Service and pharmaceutical manufacturer.^20^ Moreover, the types of MEA applications are heterogeneous among various countries, though the reasons for different choices are still unclear.^21^ The objective of this observational study is to examine the importance of MEAs for the sustainability of pharmaceutical spending in Italy by analyzing the financial outcomes of MEAs for medications that were monitored through individual data registries from 2019 to 2021.

## Methods

In this observational study, we included medications that generated a payback between 2019 and 2021 and were monitored through individually collected data registries in the analysis. Only medications that were marketed during the 3-year study period and reimbursed by INHS in December 2021 were included. Medications that were monitored in registries that closed within the study period were excluded, as complete data for the period was unavailable for those products. Medications not reimbursed or not delivered within the INHS were also excluded. This study was exempt from institutional review board approval because only aggregate economic data, not patient characteristics, were analyzed.

Paybacks from manufacturers to payers for each medication and type of MEA in the data analysis were monitored through the pharmaceutical spending monitoring activities conducted by AIFA. The expenditure data from 2019 to 2021 were collected through a national administrative database, the Italian Drug Traceability System, to which manufacturers provide sales data on pharmaceuticals purchased by all public health facilities. Expenditures were based on the purchase price truly paid by public health facilities (ie, net of confidential discounts). The expenditure data for medications were exhibited with all therapeutic indications included in a single presentation (ie, the expenditure values were not available by indication). In the case of a medication with multiple reimbursed indications, only some of which may be subject to an MEA, the payback was compared with the overall expenditure for the related medicinal product. A sensitivity analysis was conducted including a small sample of medications for which expenditure values by indication were available.

Each medication was grouped according to one of 3 MEA types: financial-based, outcome-based, or mixed for medications that have multiple indications with different MEA types. Financial-based MEAs are further divided into 2 subtypes, cost sharing and capping MEAs. Cost-sharing MEAs provide discounts on the price of the first course or total duration of therapy for all treatment-eligible patients. This tool is usually implemented when the financial outcomes and clinical efficacy of a medicine are uncertain. Capping MEAs require the manufacturer to generate paybacks when the doses or number of cycles per patient defined at negotiation are exceeded. In addition, outcome-based MEAs are also divided into 2 subtypes, risk-sharing and payment-by-result MEAs. The risk-sharing model requires manufacturers to accept a discounted price for patients who do not respond to treatment, whereas the payment-by-result model requires a full refund by the pharmaceutical manufacturer for patients who do not respond to treatment. Usually, both risk-sharing MEAs and payment-by-result MEAs are implemented when the risk-benefit ratio is uncertain, and the definition of nonresponse is based on results of pivotal clinical trials.

To overcome the time misalignment between paybacks and expenditure data, the main analysis was conducted using cumulative data during the 3 years from 2019 to 2021. Payback data were measured when received from manufacturers, not when the medicine dispensation occurred, while expenditure data were collected when the medicine was purchased. The total amount of payback received for each medicine from 2019 to 2021, resulting from the application of the different MEAs, was calculated. The total amount of payback, as calculated, was compared with the expenditure on the corresponding medications observed in the same period. The proportion of payback to expenditure was calculated by type of agreement within each category of MEA. To examine the sensitivity of the findings to the potential time misalignment between paybacks and expenditures, we conducted an analysis stratifying the payback gained within a single year.

## Results

From 2019 to 2021, 73 medications with MEAs generated a payback by manufacturers; in this analysis, 11 of these medications (15%) were excluded. Six medications were not reimbursable or delivered within INHS, and 5 medications were monitored by registries closed during the reference period. The final sample for analysis included 62 medications (eTable 1, eFigure 1A in Supplement 1). A total of 53 of 62 medications (85%) belonged to the Anatomical Therapeutic Chemical (ATC) group antineoplastic and immunomodulating agents (L), and 5 of 62 medications (8%) belonged to the ATC group anti-infectives for systemic use (J), including medication for the treatment of hepatitis C (eFigure 1B in Supplement 1).

A total of 30 medications (48.4%) were subject to an outcome-based MEA, while 24 medications (38.7%) had a financial-based MEA. Only 8 medications (12.9%) were affiliated with mixed MEAs (Table).

**Table.** Number of Medicinal Products, Overall Expenditure, Payback Amount, and Proportion of Payback to Expenditures by Category of Agreement and Subtype^a^

Category of agreement/subtype	2019-2021			
	Medicinal products, No. (%)^b^	Overall expenditure, €	Payback, € (%)	Median payback on expenditure, %
Financial-based MEAs	24 (38.7)	5 181 664 024	158 145 261 (48.3)	3.7
Capping	4 (6.5)	1 324 160 430	13 373 625 (4.1)	1.9
Capping/cost sharing	1 (1.6)	105 068 629	11 449 359 (3.5)	10.9
Cost sharing	19 (30.6)	3 752 434 965	133 322 277 (40.7)	3.7
Health outcome–based MEAs	30 (48.4)	2 498 959 121	74 494 328 (22.7)	3.3
Payment by result	28 (45.2)	2 293 803 153	72 352 104 (22.1)	4.4
Payment by result/risk sharing	2 (3.2)	205 155 968	2 142 223 (0,7)	1.0
Mixed MEAs	8 (12.9)	1 189 667 550	94 871 381 (29.0)	6.7
Capping/cost sharing/payment by result	1 (1.6)	351 573 528	51 897 706 (15.8)	14.8
Cost sharing/payment by result	7 (11.3)	838 094 022	42 973 675 (13.1)	6.6
Total	62	8 870 290 695	327 510 970	3.8

Abbreviation: MEA, managed entry agreement.

^a^ Total pharmaceutical expenditures purchased by public health facilities in Italy amounted to €41.1 billion (US $45.0 billion) from 2019 to 2021.

^b^ Financial outcomes of MEAs associated with medicinal products in data registries that were closed within the 3-year reference period (2019 to 2021) were excluded, as the data were incomplete. Medicinal products not reimbursable or delivered within the Italian National Health Service were also excluded from the analysis.

A total payback amount of €327.5 million (US $358.5 million) was received from the sample medications during the 3 years, corresponding to 0.9% of the €41.1 billion (US $45.0 billion) total expenditures for medications purchased by public health facilities in Italy. Although medicinal products were more frequently subject to outcome-based MEAs than to financial-based MEAs, the largest share of paybacks, €158.1 million (US $173.1 million), resulted from financial-based MEAs, whereas the outcome-based MEAs generated €74.5 million (US $81.6 million) in paybacks. Mixed MEAs generated €94.9 million (US $103.8 million).

The median (IQR) overall proportion of payback to expenditure for the medications included in the analysis was 3.8% (0.5%-8.3%). For mixed MEAs, the median (IQR) proportion of payback to expenditure (hereafter, payback share) was 6.7% (2.1%-9.5%). For outcome-based MEAs, the median (IQR) payback share was 3.3% (0.5%-7.5%), and for financial-based MEAs, the median (IQR) payback share was 3.7% (0.6%-9.6%) (Table). Within each type of MEA, payback share varied (Figure). The maximum values for payback share were 15% for mixed MEAs, 17% for outcome-based MEAs, and 33% for financial-based MEAs.

**Figure.**
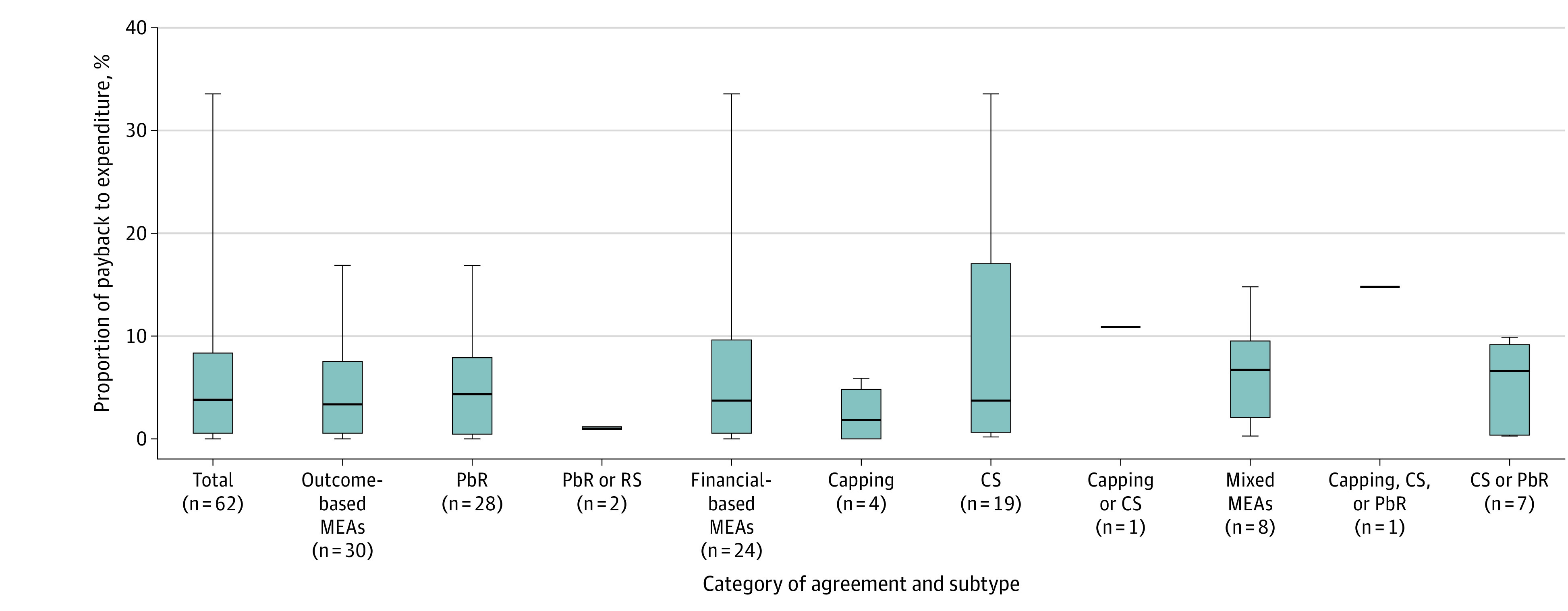
Distribution of Proportions of Payback Values to Total Expenditures by Category of Agreement and Subtype In this box plot, the lines splitting the boxes represent the median payback shares as a proportion of expenditures. The lower edge of the box represents the lower quartiles of payback share values by category of agreement and subtype. The upper edge of the box represents the upper quartile. At the end of the whiskers, the minimum and maximum payback share values are represented by category and subtype. CS indicates cost sharing; MEA, managed entry agreement; PbR, payment by result; RS, risk sharing.

In a sensitivity analysis, the payback share was calculated in 7 of the medications for which the expenditure data were available by therapeutic indication. For outcome-based MEAs, the median payback share was 4.8%; for financial-based MEAs, the payback share was 2.0% (eTable 2 in Supplement 1). These results are largely consistent with the main analysis.

In this analysis, 50 of 62 medications presented a payback share less than 10%. The majority of medications with a payback share greater than 10% had expenditure values less than the median of €65.0 million (US $71.2 million), and most medications with payback shares lower than 5% were those with higher expenditure (eFigure 2 in Supplement 1).

Another sensitivity analysis stratifying the payback gained in a single year yielded similar results to the main analysis. The numbers of medicinal products generating revenues in 2019, 2020, and 2021 were 54, 55, and 59, respectively. The median (IQR) payback share for all agreements was 3.6% (0.9%-9.9%) in 2019, 4.7% (0.9%-11.4%) in 2020, and 2.7% (0.4%-10.2%) in 2021 (eTables 3, 4, and 5; eFigures 3, 4, and 5 in Supplement 1).

## Discussion

MEAs have been implemented in several countries worldwide to manage uncertainties around the added therapeutic value of medications. Nevertheless, little is known about the financial benefits of such arrangements. A recent report from the World Health Organization cast doubt on the effectiveness of MEAs in achieving affordable and equitable access to novel medicines.^22^ In our observational study, we quantified the magnitude of payments from manufacturers in different types of MEAs (either financial-based, outcome-based, or mixed) in Italy, an early adopter with the highest number of MEAs. Overall, the median payback share was 3.8% of expenditures for the medications included in the sample (3.7% for financial-based schemes, 3.3% for outcome-based schemes, and 6.7% for mixed schemes). Moreover, we demonstrated that payback shares greater than 10% were concentrated on medications with lower sales, and most medications with payback shares less than 5% were those with higher sales. In Belgium and Sweden, similar studies have been performed.^23,24,25^ However, comparisons to our findings would be limited because these studies included different medications and types of MEAs. Indeed, our study only refers to MEAs based on registries; therefore, evaluation of MEAs using price-volume agreements with or without ceiling and confidential discounts were excluded. Moreover, these other studies did not quantify the magnitude of paybacks for the medications subject to MEAs by type of agreement.

The size of paybacks resulting from financial-based MEAs appeared to be modest. We suggest comparing the magnitude of paybacks with the resources required to implement and maintain financial-based MEAs. Implementation costs include the administrative burden for health care professionals who are required to provide registries with data. Paybacks may be obtained at a later stage (ie, several months after the reimbursement decision) compared with a simple immediate discount agreed on at the time of the reimbursement decision. In addition because of their smaller paybacks, payers may reconsider the value of outcome-based MEAs. We recommend assessing the level of clinical evidence maturity and the challenges of implementing registries that capture meaningful data on health outcomes, particularly shortly after a medicine is approved when evidence to inform the choice of an appropriate threshold for patient response may be limited.^1,26,27,28,29^

The identification of the clinical threshold, outcome measure, and assessment time is crucial with consequences for the magnitude of the payback. Clear definitions of these important elements should be part of the general guidance. We suggest these definitions be detailed in the MEA and explained in the clinical guidance. Our findings on small paybacks from MEAs suggest that payers may need to identify criteria to inform the adoption of MEAs, as well as which type, to ensure its optimal use.

Moreover, we recommend that the clinical uncertainties identified at the time of approval should be resolved in a defined period of 3 to 5 years. This parameter aims to provide timely definitions of the medication’s added therapeutic value and return the collected information to clinical practice, as the evidence gaps to be filled may also change over time.^28^ An evaluation of MEAs signed in Belgium between 2010 and 2015 found no evidence within the framework of MEAs related to the research questions identified at the time of the Health Technology Assessment evaluation,^27^ suggesting that other mechanisms beyond MEAs are needed to address evidence gaps. Therefore, we suggest that MEAs have a defined duration and re-evaluations of added therapeutic value be planned to ensure timely responses to changes in the body of evidence. Moreover, periodic revisions of MEAs’ terms, leading to updates in the clinical threshold defining treatment response, may translate into better performance in terms of the payback gained. Pauwels et al^26^ found that the durations of a large number of MEAs in Europe were extended after expiration. To our knowledge in Italy, the contract generally has a 24-month duration, and the identification of cases where data registries have led to a change in reimbursability remains an open issue.

The currently available evidence suggests that MEAs may be more likely to ensure access and optimal use,^29,30^ rather than guarantee the sustainability of pharmaceutical expenditures. When choosing whether and how to implement MEAs, payers may consider the following factors: administrative burden required for implementation, which includes developing and maintaining patient registries; predictability of net expenditures associated with delayed paybacks; level of evidence available to establish thresholds for treatment response in outcome-based MEAs; duration of MEAs; and time required for data collection.

### Limitations

First, expenditure data by indication for all medications in the analysis were unavailable. Nevertheless, the sensitivity analyses on a limited sample of medications, for which expenditure data by indication (and related paybacks) were available, also demonstrated small paybacks from these MEAs. A second limitation of this study is the temporal misalignment of payback revenues and expenditure data, although sensitivity analyses conducted within calendar years were consistent with the results conducted using the 3-year cumulative data. Third, we were unable to estimate savings associated with reduced treatment failure rates obtained by the application of reimbursement criteria. These criteria, applied through the registries tool, allow the identification of patients most likely to benefit according to the available evidence. It is not possible to assess these savings in Italy because only patients meeting reimbursement criteria can access the treatment.

## Conclusions

Despite the increasing use of MEAs, scarce evidence exists regarding the potential for this instrument to manage increasing pharmaceutical expenditures. This observational study found limited evidence that MEAs lower pharmaceutical expenditures. These findings suggest the need to identify criteria for prioritizing the use of MEAs and potential changes to their design and implementation.

## Notes

Supplement 1. **eTable 1.** Medicine included in the analysis (n=62) **eFigure 1.** A, Flow-chart of medicines included in the analysis; B, Distribution of medicines included in the analysis by ATC I **eTable 2.** Number of medicinal products, amount of payback and payback share by type and category of agreement in 2019-2021; only medicine with expenditure data by indication were included **eFigure 2.** Relation between payback share and INHS expenditure 2019-2021 **eTable 3.** Number of medicinal products, overall expenditure, amount of payback and payback share on expenditure by type and category of agreement in 2019 **eFigure 3.** Box plot: share of payback on expenditure by type and category of agreement: year 2019 **eTable 4.** Number of medicinal products, overall expenditure, amount of payback and payback share on expenditure by type and category of agreement in 2020 **eFigure 4.** Box plot: distribution of the magnitude of paybacks shown as share of total expenditures, by type and category of agreement: year 2020 **eTable 5.** Number of medicinal products, overall expenditure, amount of payback and payback share on expenditure by type and category of agreement in 2021 **eFigure 5.** Box plot: distribution of the magnitude of paybacks shown as share of total expenditures, by type and category of agreement: year 2021

Supplement 2.
Data Sharing Statement
