# Supplementary material for: Financial Outcomes of Managed Entry Agreements for Pharmaceuticals in Italy
Source: JAMA Health Forum. 2023 Dec 28;4(12):e234611. doi: 10.1001/jamahealthforum.2023.4611 (PMC10755625; doi:10.1001/jamahealthforum.2023.4611)
Supplement: Supplement 1. — eTable 1. Medicine included in the analysis (n=62) eFigure 1. A, Flow-chart of medicines included in the analysis; B, Distribution of medicines included in the analysis by ATC I eTable 2. Number of medicinal products, amount of payback and payback share by type and category of agreement in 2019-2021; only medicine with expenditure data by indication were included eFigure 2. Relation between payback share and INHS expenditure 2019-2021 eTable 3. Number of medicinal products, overall expenditure, amount of payback and payback share on expenditure by type and category of agreement in 2019 eFigure 3. Box plot: share of payback on expenditure by type and category of agreement: year 2019 eTable 4. Number of medicinal products, overall expenditure, amount of payback and payback share on expenditure by type and category of agreement in 2020 eFigure 4. Box plot: distribution of the magnitude of paybacks shown as share of total expenditures, by type and category of agreement: year 2020 eTable 5. Number of medicinal products, overall expenditure, amount of payback and payback share on expenditure by type and category of agreement in 2021 eFigure 5. Box plot: distribution of the magnitude of paybacks shown as share of total expenditures, by type and category of agreement: year 2021 [file jamahealthforum-e234611-s001.pdf]

Supplemental Online Content

Trotta F, Guerrizio MA, Di Filippo A, Cangini A. Financial outcomes of managed entry agreements for pharmaceuticals in Italy. *JAMA Health Forum.* 2023;4(12):e234611. doi:10.1001/jamahealthforum.2023.4611

eTable 1. Medicine included in the analysis (n=62)

eFigure 1. A, Flow-chart of medicines included in the analysis; B, Distribution of medicines included in the analysis by ATC I

eTable 2. Number of medicinal products, amount of payback and payback share by type and category of agreement in 2019-2021; only medicine with expenditure data by indication were included

eFigure 2. Relation between payback share and INHS expenditure 2019-2021

eTable 3. Number of medicinal products, overall expenditure, amount of payback and payback share on expenditure by type and category of agreement in 2019

eFigure 3. Box plot: share of payback on expenditure by type and category of agreement: year 2019

eTable 4. Number of medicinal products, overall expenditure, amount of payback and payback share on expenditure by type and category of agreement in 2020

eFigure 4. Box plot: distribution of the magnitude of paybacks shown as share of total expenditures, by type and category of agreement: year 2020

eTable 5. Number of medicinal products, overall expenditure, amount of payback and payback share on expenditure by type and category of agreement in 2021

eFigure 5. Box plot: distribution of the magnitude of paybacks shown as share of total expenditures, by type and category of agreement: year 2021

This supplemental material has been provided by the authors to give readers additional information about their work.

© 2023 Trotta F et al. *JAMA Health Forum.*

eTable 1. Medicine included in the analysis (n=62)

ATC	Active ingredient
L01CD01	PACLITAXEL
L01FX05	BRENTUXIMAB VEDOTIN
L01EG02	EVEROLIMUS
L01ED03	ALECTINIB
L01BA04	PEMETREXED
L01FG01	BEVACIZUMAB
L01FX07	BLINATUMOMAB
L01EA04	BOSUTINIB
L01EX04	VANDETANIB
L01EE02	COBIMETINIB
M05BX05	BUROSUMAB
L01FG02	RAMUCIRUMAB
L01BC08	DECITABINA
L01FC01	DARATUMUMAB
J05AP55	SOFOSBUVIR/VELPATASVIR
L01FE01	CETUXIMAB
L01XJ01	VISMODEGIB
L01FA03	OBINUTUZUMAB
L01EB03	AFATINIB
L01XX41	ERIBULINA
L01FD01	TRASTUZUMAB
L04AB04	ADALIMUMAB
L01EA05	PONATINIB
L01EK01	AXITINIB
L01EB01	GEFITINIB
L01EJ01	RUXOLITINIB
L01CA05	VINFLUNINA
L01FD03	TRASTUZUMAB EMTANSINE
L01XG02	CARFILZOMIB
S01LA04	RANIBIZUMAB
J05AP57	GLECAPREVIR/PIBRENTASVIR
L01EE01	TRAMETINIB
L03AX16	PLERIXAFOR
L01EX02	SORAFENIB
L01FF01	NIVOLUMAB
L04AX04	LENALIDOMIDE
N02BG10	DELTA-9-TETRAIDROCANNABINOLO/CANNABIDIOLO
H01CB05	PASIREOTIDE
L04AB06	GOLIMUMAB
J04AK05	BEDAQUILINA
L01EA02	DASATINIB
L01EX01	SUNITINIB
L01EC02	DABRAFENIB
L01EB04	OSIMERTINIB

© 2023 Trotta F et al. *JAMA Health Forum.*

L01EB02	ERLOTINIB
L01EA03	NILOTINIB
L01EH01	LAPATINIB
L01EX09	NINTEDANIB
L01FE02	PANITUMUMAB
L01XG01	BORTEZOMIB
L01BC07	AZACITIDINA
J05AP56	SOFOSBUVIR/VELPATASVIR/VOXILAPREVIR
L01EX03	PAZOPANIB
L01ED01	CRIZOTINIB
L02BB04	ENZALUTAMIDE
L01FX04	IPILIMUMAB
L01CX01	TRABECTEDINA
L01XX44	AFLIBERCEPT
L01EC01	VEMURAFENIB
J05AP54	ELBASVIR/GRAZOPREVIR
L01ED02	CERITINIB
L02BX03	ABIRATERONE

© 2023 Trotta F et al. *JAMA Health Forum.*

eFigure 1A. Flow-chart of medicines included in the analysis

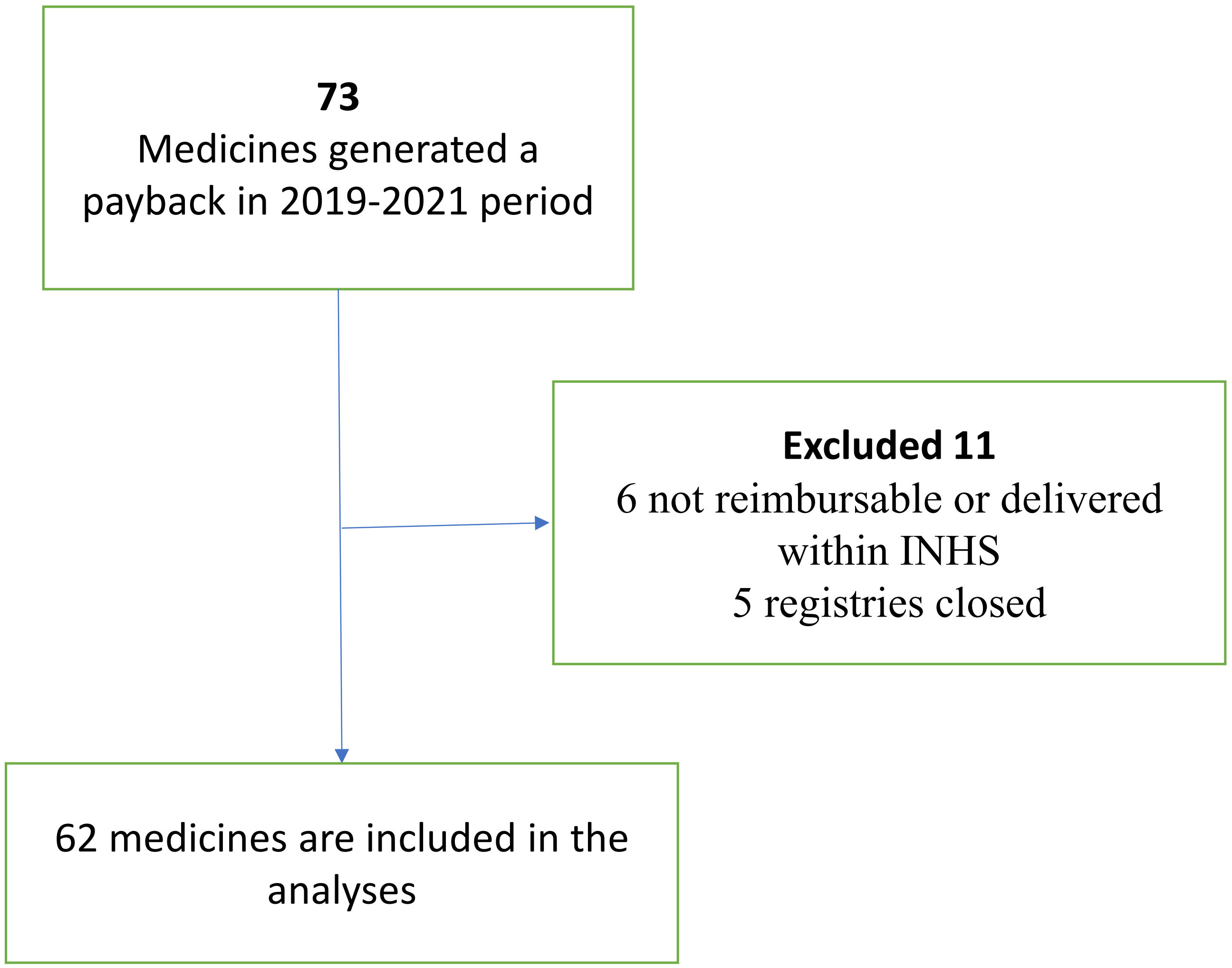

© 2023 Trotta F et al. *JAMA Health Forum.*

eFigure 1B. Distribution of medicines included in the analysis by ATC I

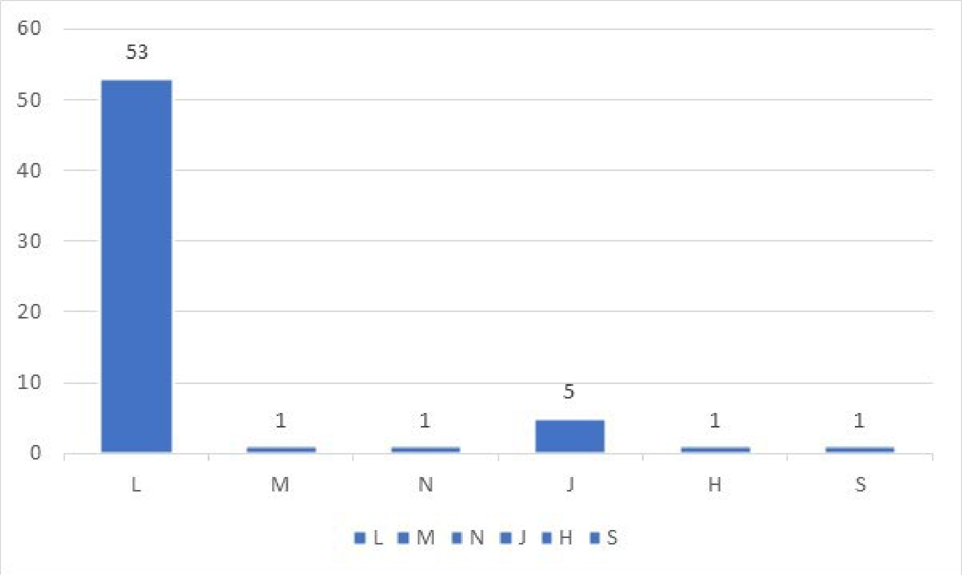

Table e2. Number of medicinal products, amount of payback and payback share by type and category of agreement in 2019-2021; only medicine with expenditure data by indication were included.

Type of agreement/category	Number of medicinal products	Pay-back 2019-2021 (euro)	Payback % on expenditure (median)
**MEA Outcome-based**	**3**	**15.480.784,3**	**4,8%**
Payment by result	3	15.480.784,3	4,8%
**MEA Financia- based**	**4**	**26.141.791,9**	**2,0%**
Cost-sharing	3	13.437.402,2	1,2%
Capping	1	12.704.389,7	5,9%
**Total**	**7**	**41.622.576,3**	**2,6%**

Medicines included: dabrafenib, trametinib, osimertinib, daratumumab, elbasvir/grazoprevir, alectinib, glecaprevir/pibrentasvir

eFigure 2. Relation between payback share and INHS expenditure 2019-2021

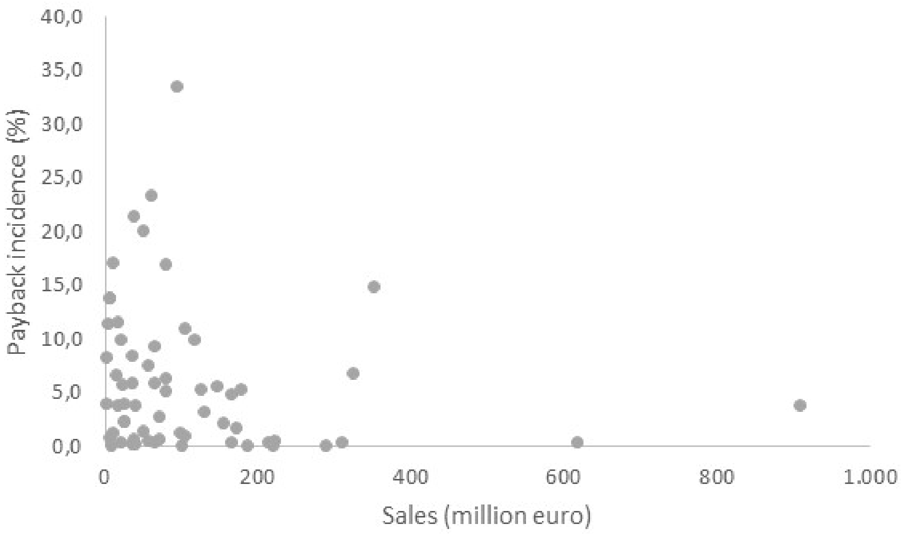

© 2023 Trotta F et al. *JAMA Health Forum.*

eTable 3. Number of medicinal products, overall expenditure, amount of payback and payback share on expenditure by type and category of agreement in 2019

Type of agreement/Category	Number of medicinal products N(%)	Overall expenditure 2019 (euro)	Pay-back 2019 euro (%)	Payback % on expenditure (median)
**MEA financial-based**	**18 (33.3%)**	**1.035.967.871**	**35.038.878 (34.1%)**	**2.8**
Capping	1 (1.9%)	124.724.901	4.824.632 (4.7%)	3.9
Capping/Cost-sharing	1 (1.9%)	34.680.808	677.947 (0.7%)	2.0
Cost-sharing	16 (29.6%)	876.562.162	29.536.299 (28.8%)	2.8
**MEA Outcome-based**	**29 (53.7%)**	**817.799.783**	**29.573.429 (28.8%)**	**3.3**
Payment by result /Risk-sharing	2 (3.7%)	69.520.191	1.586.187 (1.5%)	2.3
Payment by result	27 (50.0%)	748.279.592	27.987.242 (27.3%)	5.6
**MEA mixed**	**7 (13.0%)**	**410.344.022**	**38.040.817 (37.1%)**	**6.6**
Capping/Cost-sharing/Payment by result	1 (1.9%)	193.554.392	18.955.376 (18.5%)	9.8
Cost-sharing /Payment by result	6 (11.1%)	216.789.629	19.085.441 (18.6%)	5.6
**TOTAL**	**54 (100%)**	**2.264.111.678**	**102.653.124 (100%)**	**3.6**

© 2023 Trotta F et al. *JAMA Health Forum.*

eFigure 3. Box plot: share of pay-back on expenditure by type and category of agreement: year 2019

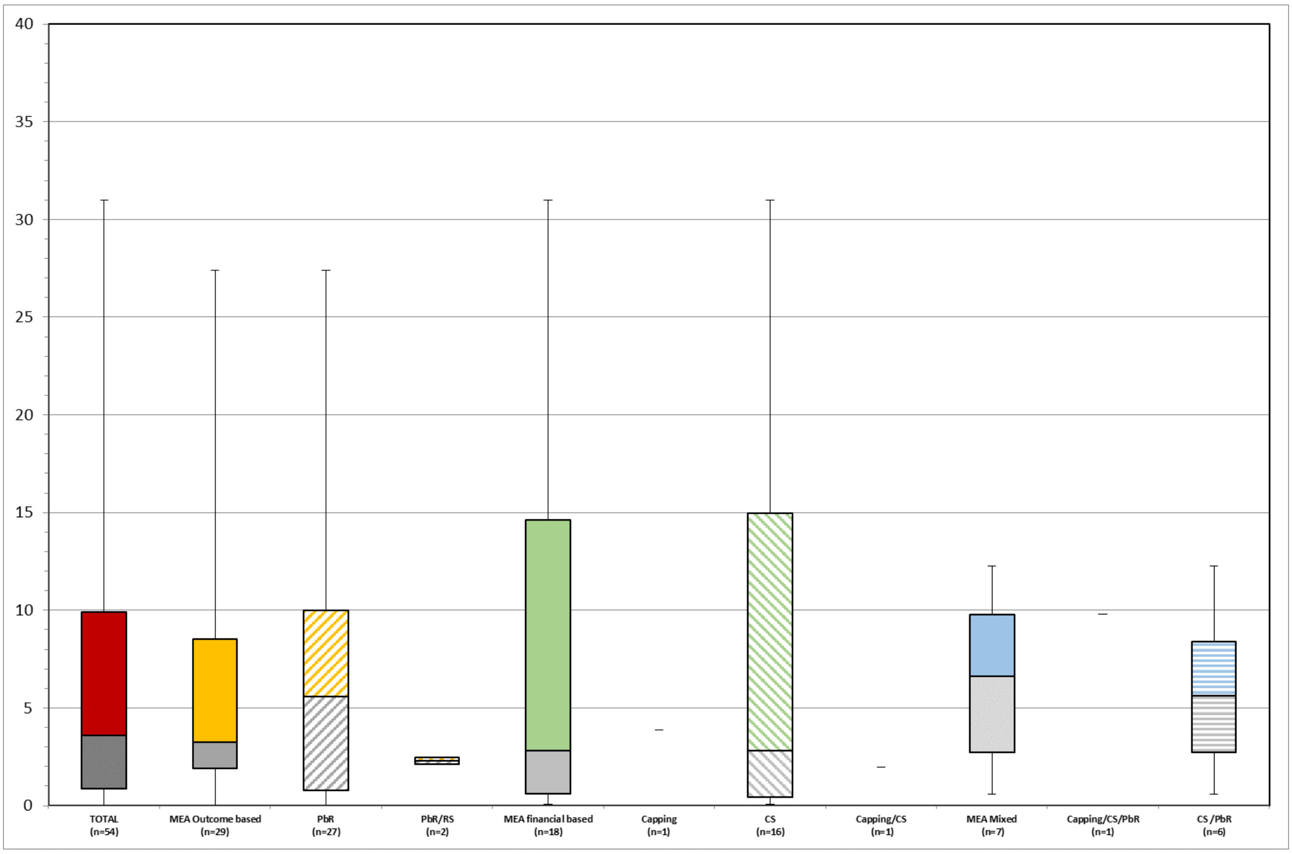

PbR: Payment by Result RS: Risk sharing CS: Cost-sharing

Note: The line splitting the box in two represents the median value. The lower edge of the box represents the lower quartile. The upper edge of the box shows the upper quartile; at the end of the whiskers the minimum and maximum values are represented.

© 2023 Trotta F et al. *JAMA Health Forum.*

eTable 4. Number of medicinal products, overall expenditure, amount of payback and payback share on expenditure by type and category of agreement in 2020

Type of agreement/Category	Number of medicinal products N (%)	Overall expenditure 2020 (Euro)	Pay-back 2020 Euro (%)	Payback Incidence% on expenditure (median)
**MEA Financial based**	**19 (34.5%)**	**1.128.751.829**	**49.880.408 (47.6%)**	**7.3**
Capping	1 (1.8%)	50.155.251	5.282.003 (5.0%)	10.5
Capping/Cost-sharing	1 (1.8%)	35.739.042	3.228.698 (3.1%)	9.0
Cost-sharing	17 (30,9%)	1.042.857.535	41.369.706 (39.5%)	6.2
**MEA Outcome based**	**28 (50.9%)**	**786.208.625**	**28.734.949 (27.4%)**	**4.1**
Payment by result	26 (47.3%)	717.120.223	28.329.842 (27.0%)	4.5
Payment by result /Risk-sharing	2 (3.6%)	69.088.402	405.106,53 (0.4%)	0.6
**MEA Mixed**	**8 (14.5%)**	**414.037.568**	**26.130.517 (24.9%)**	**5.3**
Capping/Cost-sharing/Payment by result	1 (1.8%)	131.902.657	15.023.828 (14.3%)	11.4
Cost-sharing /Payment by result	7 (12.7%)	282.134.911	11.106.689 (10.6%)	3.9
**TOTAL**	**55 (100.0%)**	**2.328.998.021**	**104.745.874,45 (100.0%)**	**4.7**

© 2023 Trotta F et al. *JAMA Health Forum.*

eFigure 4. Box plot: distribution of the magnitude of paybacks shown as share of total expenditures, by type and category of agreement: year 2020

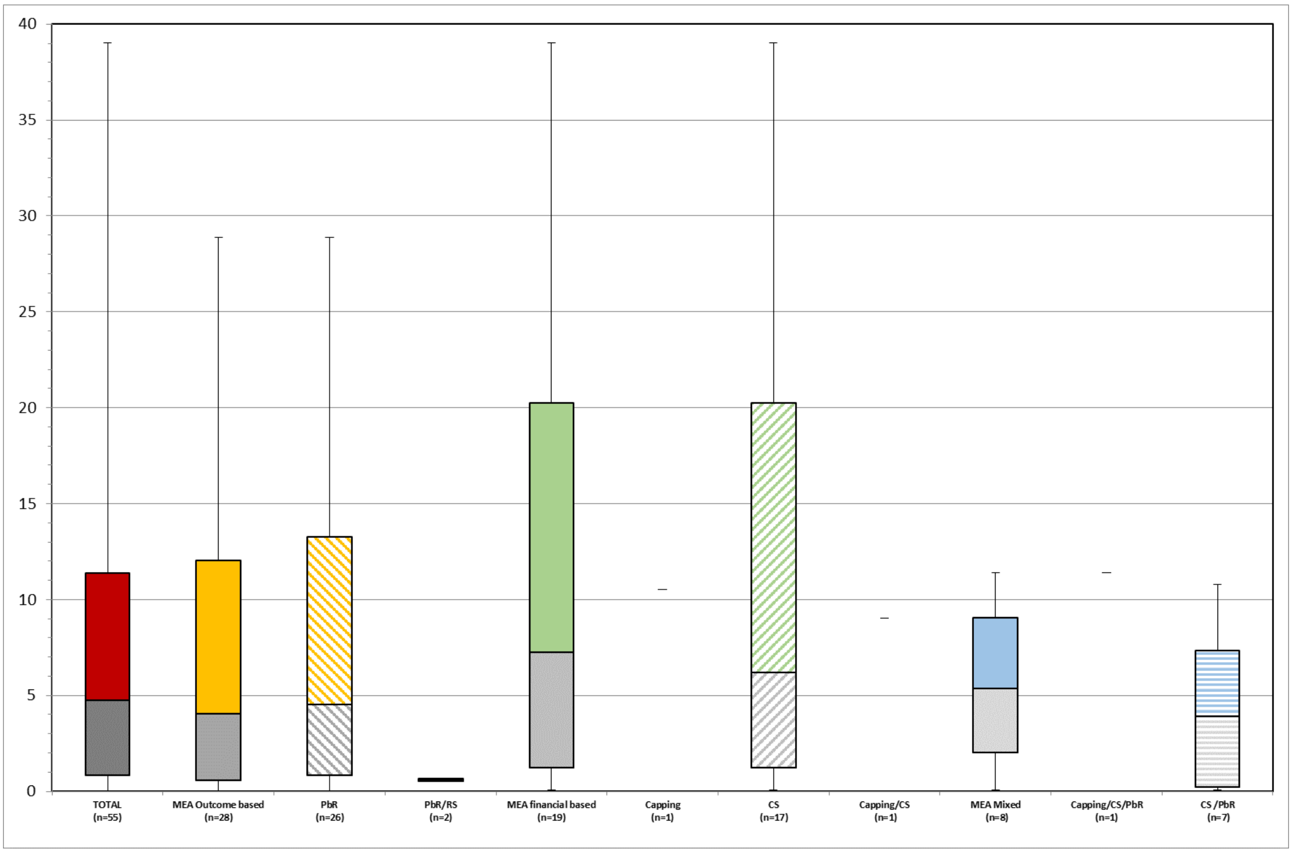

PbR: Payment by Result RS: Risk sharing CS: Cost-sharing

Note: The line splitting the box in two represents the median value. The lower edge of the box represents the lower quartile. The upper edge of the box shows the upper quartile; at the end of the whiskers the minimum and maximum values are represented.

© 2023 Trotta F et al. *JAMA Health Forum.*

eTable 5. Number of medicinal products, overall expenditure, amount of payback and payback share on expenditure by type and category of agreement in 2021

Type of agreement/Category	Number of medicinal products N (%)	Overall expenditure (euro)	Pay-back 2021 euro (%)	Payback % on expenditure (median)
**MEA Financial-based**	**23 (39.0%)**	**1.412.619.200**	**73.314.921 (61.1%)**	**3.0**
Capping	4 (6.8%)	93.819.60	3.266.989 (2.7%)	2.9
Capping/Cost-sharing	1 (1.7%)	34.648.778	7.542.714 (6.3%)	21.8
Cost-sharing	18 (30.5%)	1.284.150.815	62.505.218 (52.0%)	2.8
**MEA Outcome-based**	**28 (47.5%)**	**742.382.823**	**16.185.949 (13.5%)**	**2.0**
Payment by result	26 (44.1%)	675.835.448	16.035.020 (13.4%)	2.4
Payment by result /Risk-sharing	2 (3.4%)	66.547.375	150.929 (0.1%)	0.2
**MEA Mixed**	**8 (13.6%)**	**291.782.443**	**30.611.101 (25.5%)**	**6.9**
Cost-sharing /Payment by result	7 (11.9%)	265.665.965	12.692.599 (10.6%)	4.7
Capping/Cost-sharing /Payment by result	1 (1.7%)	26.116.478	17.918.502 (14.9%)	68.6
**TOTAL**	**59 (100.0%)**	**2.446.784.466**	**120.111.971 (100.0%)**	**2.7**

© 2023 Trotta F et al. *JAMA Health Forum.*

eFigure 5. Box plot: distribution of the magnitude of paybacks shown as share of total expenditures, by type and category of agreement: year 2021

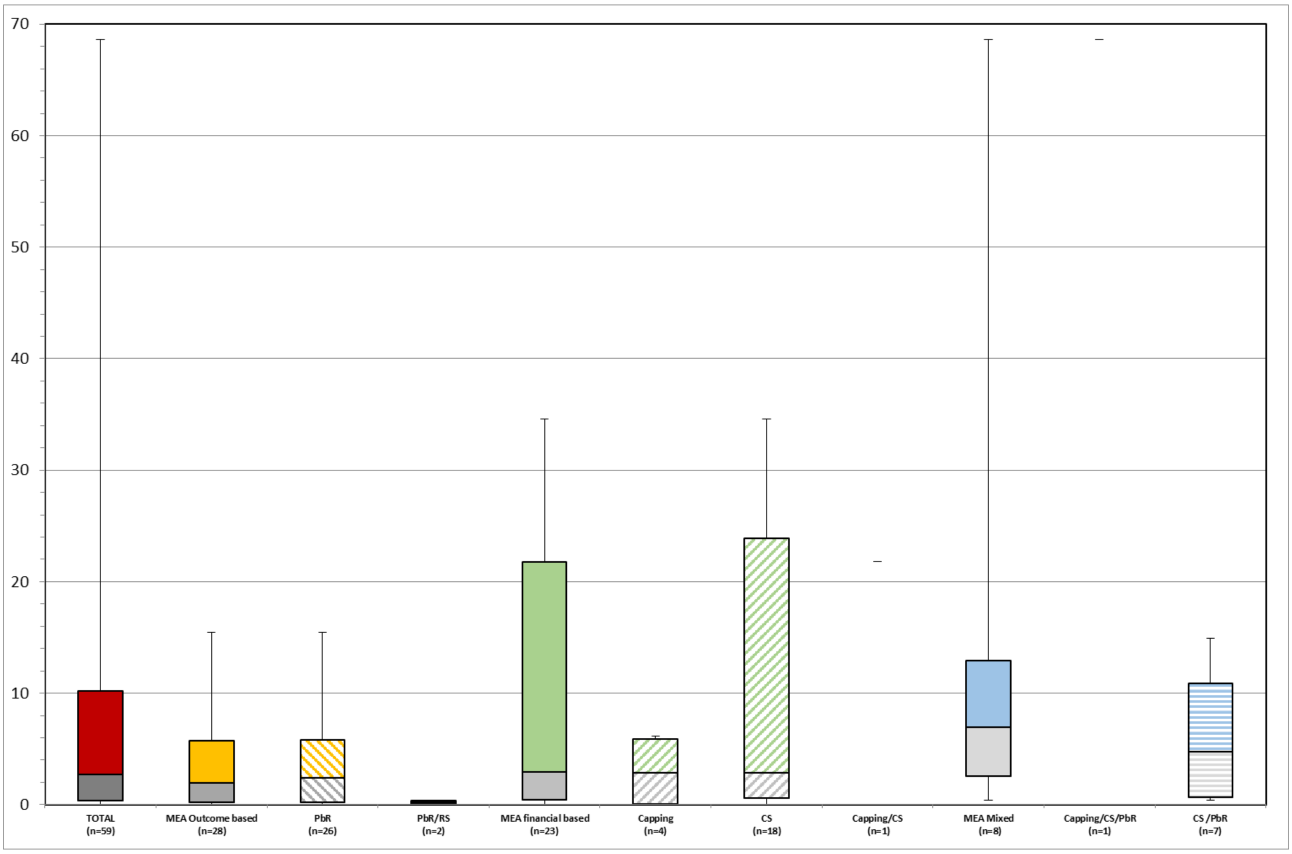

Note: The line splitting the box in two represents the median value. The lower edge of the box represents the lower quartile. The upper edge of the box shows the upper quartile; at the end of the whiskers the minimum and maximum values are represented.

© 2023 Trotta F et al. *JAMA Health Forum.*
