# Supplementary material for: Financial Outcomes of Managed Entry Agreements for Pharmaceuticals in Italy
Source: JAMA Health Forum. 2023 Dec 28;4(12):e234611. doi: 10.1001/jamahealthforum.2023.4611 (PMC10755625; doi:10.1001/jamahealthforum.2023.4611)
Supplement: Supplement 2. — Data Sharing Statement [file jamahealthforum-e234611-s002.pdf]

Data Sharing Statement

Trotta. Financial Outcomes of Managed Entry Agreements for Pharmaceuticals in Italy. *JAMA Health Forum*. Published December 28, 2023. doi:10.1001/jamahealthforum.2023.4611

Data
Data available: No

Additional Information
Explanation for why data not available: Due to confidentiality of data analysed in the study they can be shared only in aggregated format.
